# Effect of Grazing Angle Cross-Ion Irradiation on Ag Thin Films

**DOI:** 10.1186/s11671-016-1665-5

**Published:** 2016-10-11

**Authors:** Manish Kumar, Teena Jangid, Vandana Panchal, Praveen Kumar, Abhishek Pathak

**Affiliations:** 1 Department of Physics, Central University of Rajasthan, NH-8, Kishangarh, 305801 India; 2 Department of Physics, Kurukshetra University, Kurukshetra, 136119 India; 3 Inter University Accelerator Centre, Aruna Asaf Ali Marg, New Delhi, 110067 India; 4 Ajay Kumar Garg Engineering College, Adhyatmik Nagar, Ghaziabad, 201009 India

**Keywords:** Sputtering, Sputter re-deposition, Silver, Irradiation, Cone-like nanostructures

## Abstract

Apart from the spherical shape, control over other shapes is a technical challenge in synthesis approaches of nanostructures. Here, we studied the effect of grazing angle cross-irradiation Ag thin films for the nanostructures evolution from a top-down approach. Ag thin films of different thicknesses were deposited on Si (100) and glass substrates by electron beam evaporation system and subsequently irradiated at grazing angle ions by 80 keV Ar^+^ in two steps (to induce effectively a cross-ion irradiation). Pristine films exhibited dense and uniform distribution of Ag nanoparticles with their characteristic surface plasmon resonance-induced absorption peak around 420 nm. When the film surfaces were treated with cross-grazing angle irradiation of Ar ions with varying effective fluences from 0.5 × 10^17^ ions/cm^2^ to 2.0 × 10^17^ ions/cm^2^, it was found that fluence values governed the competition of sputtering and sputter re-deposition of Ag. As a result, lower irradiation fluence favoured the formation of cone-like nanostructures, whereas high fluence values demonstrated dominant sputtering. Fluence-dependent modification of surface features was studied through the Fourier transform infrared spectroscopy and the Rutherford backscattering spectroscopy. Theoretical justifications for the underlying mechanisms are presented to justify the experimental results.

## Background

Ag nanostructures, owing to the strong coupling of surface plasmons with the incident light, exhibit interesting properties, e.g. photochemical activity [1], antibacterial activity [2], linear [3] and nonlinear optical properties [4] and surface-enhanced Raman scattering [5]. Because the surface plasmons in metal nanostructures are highly sensitive to the subtle difference in their shape, size, distribution and ambient matrix, the morphology of Ag nanostructures strongly governs all these forementioned properties. For exploring the new possibilities in tailored nanostructures, researchers have attempted nanocups [6], nanobars and nanorice [7], helical and multi-ring [8], nanoflowers [9], nanoplates [10, 11] and nanocones [12–16] which are kinds of Ag nanostructures. Even the roughened Ag surfaces were found to be exciting for surface-enhanced properties [17, 18]. Theoretically, works predicted particularly cone/tip-like structures as important optical antennas for near-field microscopy [15] and also for the molecular plasmonics where it is sought to have full control on the interaction between confined plasmons and point sources of radiation [19, 20].

As a synthesis methodology, low energy ion irradiation on a surface can result in self-organized nanoscale periodic patterns on solid surfaces [21]. The gradient of the atomic flux parallel to the surface, generated by ion bombardment may lead to net smoothening for normal incidence conditions, whereas for larger incidence angles, roughening and ripple formation can occur [21–24]. In this way, sputtering of the ion beam sometime results in a smooth surface and some time in a rough surface. Under certain conditions, irradiation may also induce well-ordered topographies, like ripples [22–25], dots [26], cones [27] and nanowires [28]. These nanoscale periodic structures have found many potential applications. For example, ripple patterns have found applications in plasmonics and nanoscale magnetism as templates for growth of functional thin films [28, 29], whereas nanodot- and nanowire-type arrays are reported for fabricating trace gas sensors and opto-electronic devices, where a high degree of in-plane ordering is required [30]. Surface patterns on noble metal surfaces can also exhibit unique optical properties like simultaneously negative permittivity and permeability to be used to fabricate clocking devices [31]. However, most of surface patterning by ion irradiation is mainly focused for periodically arranged ripples/nanodots on semiconductor and compound semiconductor surfaces. On the other hand, nanostructuring on metal surfaces are mainly attempted using focused ion beams and nanoimprinting lithography [12–16], and use of low energy ions via cross-irradiation is sparsely reported [32–34]. Joe et al. investigated the simultaneous multiple cross-ion irradiation on the Au (100) and observed the formation of square-symmetric nanoholes and nanodots [32]. Vogel et al. theoretically proposed the hexagonal, square and super-structure pattern formation on amorphous surfaces by simultaneous incidence of four identical ion beams [33]. The relevance of surface anisotropy and ion-induced surface diffusion on obtained patterns were quantified on the basis of a generalized version of the damped Kuramoto–Sivashinsky equation [33]. Kim et al. attempted the pattern evolution using cross-ion beam sputtering on Au (001), but did not observe the superposition of ripples [34]. We hypothesized that depending on the right ion specy, energy, fluence and grazing angle, cross-ion irradiation may induce cross ripples (superposition of ripples), which should effectively yield a cone/spike-like nanostructure.

This work presents the feasibility of cone-like Ag nanostructures by grazing angle cross-ion irradiation. After the Ag thin film preparation, grazing angle cross-irradiation using Ar ions was performed in sequential steps. The properties of pristine and irradiated samples and underlying mechanisms of irradiation-induced nanostructures are presented.

## Methods

### Film Synthesis

Si (100) wafers were cleaved into pieces (area 2 × 1 cm^2^) and cleaned before thin film preparation. Diluted solution of hydrofluoric acid was used to remove oxide layer from the surface of Si wafers. Then, Si substrates were further cleaned through ultrasonication in acetone and methanol for 3 min. These steps are repeated for two times for obtaining clean surfaces of Si. Ag thin film was deposited over the Si substrate using electron beam physical vapour deposition system [M/s Hind High Vacuum]. During the deposition of Ag films, base pressure was fixed as 6 × 10^−5^ Torr and working pressure was about 9 × 10^−5^ Torr. Through the digital thickness monitor, the deposition system allowed the control on the growth rate and overall thickness of thin film during the synthesis. Different sets of thin films, having thicknesses 10, 20 and 30 nm were prepared.

### Grazing Angle Cross-Ion Irradiation

The pristine Ag films were irradiated with mass separated 80 keV Ar^+^ ion beam with fluence values as 0.25 × 10^17^, 0.50 × 10^17^, 0.75 × 10^17^ and 1.0 × 10^17^ ions/cm^2^. The beams were incident at 60° angle to surface normal on the targets (grazing angle 30°). Second-step irradiation was carried out with similar fluences after the rotating of the Ag samples by 90°, and thus, effective fluences became two times as 0.5 × 10^17^, 1.0 × 10^17^, 1.5 × 10^17^ and 2.0 × 10^17^ ions/cm^2^, respectively.

### Characterization Techniques

UV-visible absorption spectra of pristine samples were recorded using a dual beam spectrophotometer (Hitachi U3300). To investigate the surface morphology, plane-view images were obtained using field emission scanning electron microscope (FESEM, *MIRA II* LMH). Energy dispersive X-ray spectroscopy (EDX) was employed using energy dispersive X-ray detector (INCA *PentaFET3*) attached with FESEM. Atomic force microscope (AFM, Veeco: *Nano Scope IIIa*) with SiN tips (nominal tip radius of <10 nm) was used to investigate the grown nanostructures under ambient conditions. In IR region (400–4000 cm^−1^), spectroscopic measurements of the pristine and irradiated Ag thin films were carried out using the Fourier transform infrared spectrophotometer (FTIR, PERKIN ELMER *Bx*). The Rutherford backscattering spectroscopy (RBS) was performed using 1.7 MV 5SDH-2 Pelletron (Tandem) accelerator with 2 MeV He^+^ ion beam.

## Results and Discussion

**Fig. 1:**
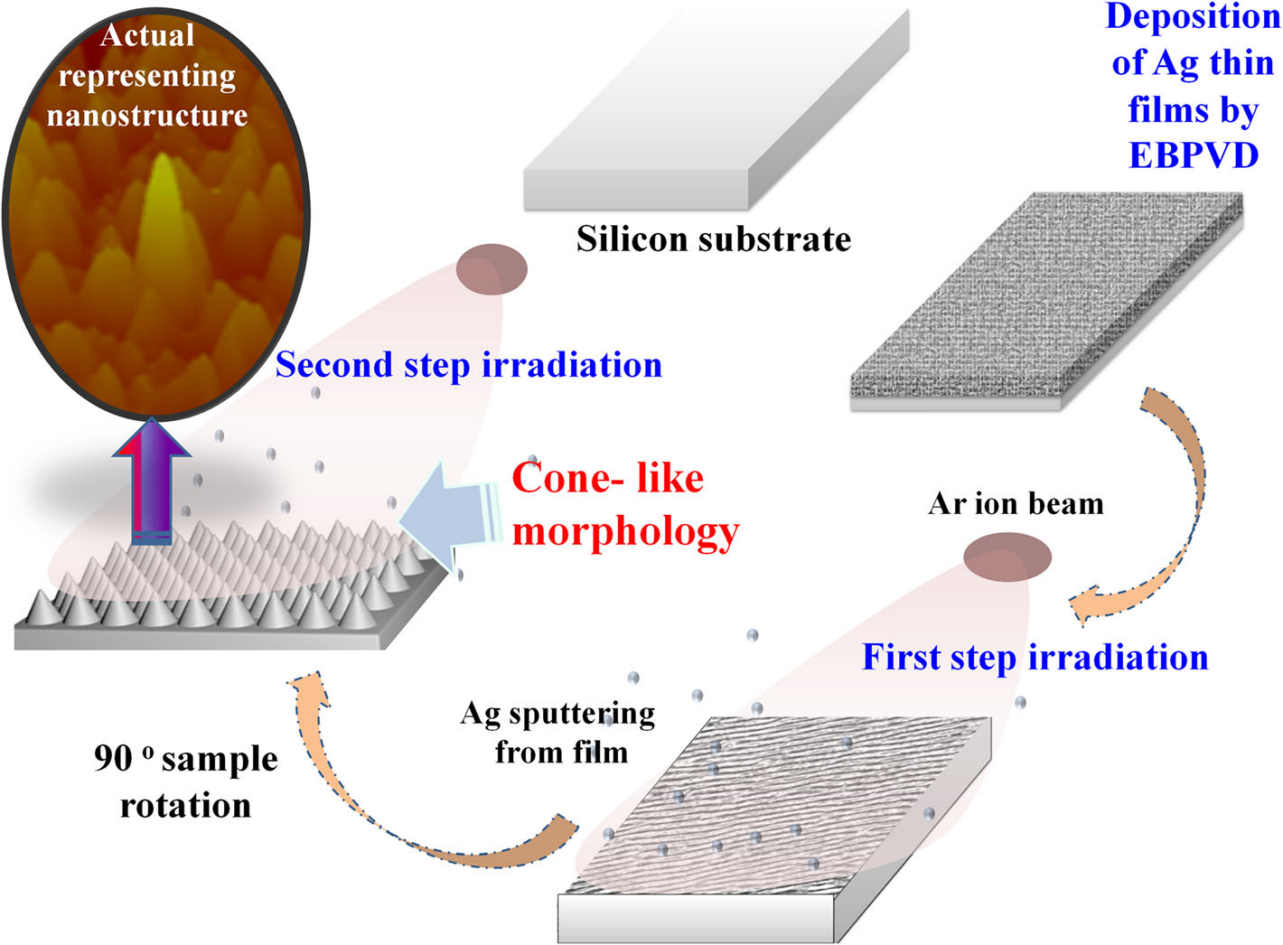
Schematic approach for cone-like structures formation using cross-angle grazing irradiation. A representing actual nanostructure is also shown in the *inset*

Figure 1 depicts the fabrication process of the cone-like nanostructure by grazing angle ion irradiation. The first step involves the deposition of the Ag thin film over the Si substrate using electron beam physical vapour deposition system. In the next step, the pristine Ag films were irradiated, at grazing angle, with mass separated 80 keV Ar^+^ ion beam with different fluence values. The incident ion beam induced the ripple-like pattern over the Ag surface. Along with the formation of the wave pattern, the sputtering of the deposited films also occurred, that is discussed in more details in further sections. Second-step irradiation was carried out with similar fluences after the rotating of the Ag samples by 90°. The rotation of the sample leads to the formation of the cone-like morphology, which is believed to be rarely reported in the literature for similar fabrication process. The details of the optical, structural and morphological changes with the change in the fabrication steps will be the subject of discussion in the further sections.

**Fig. 2:**
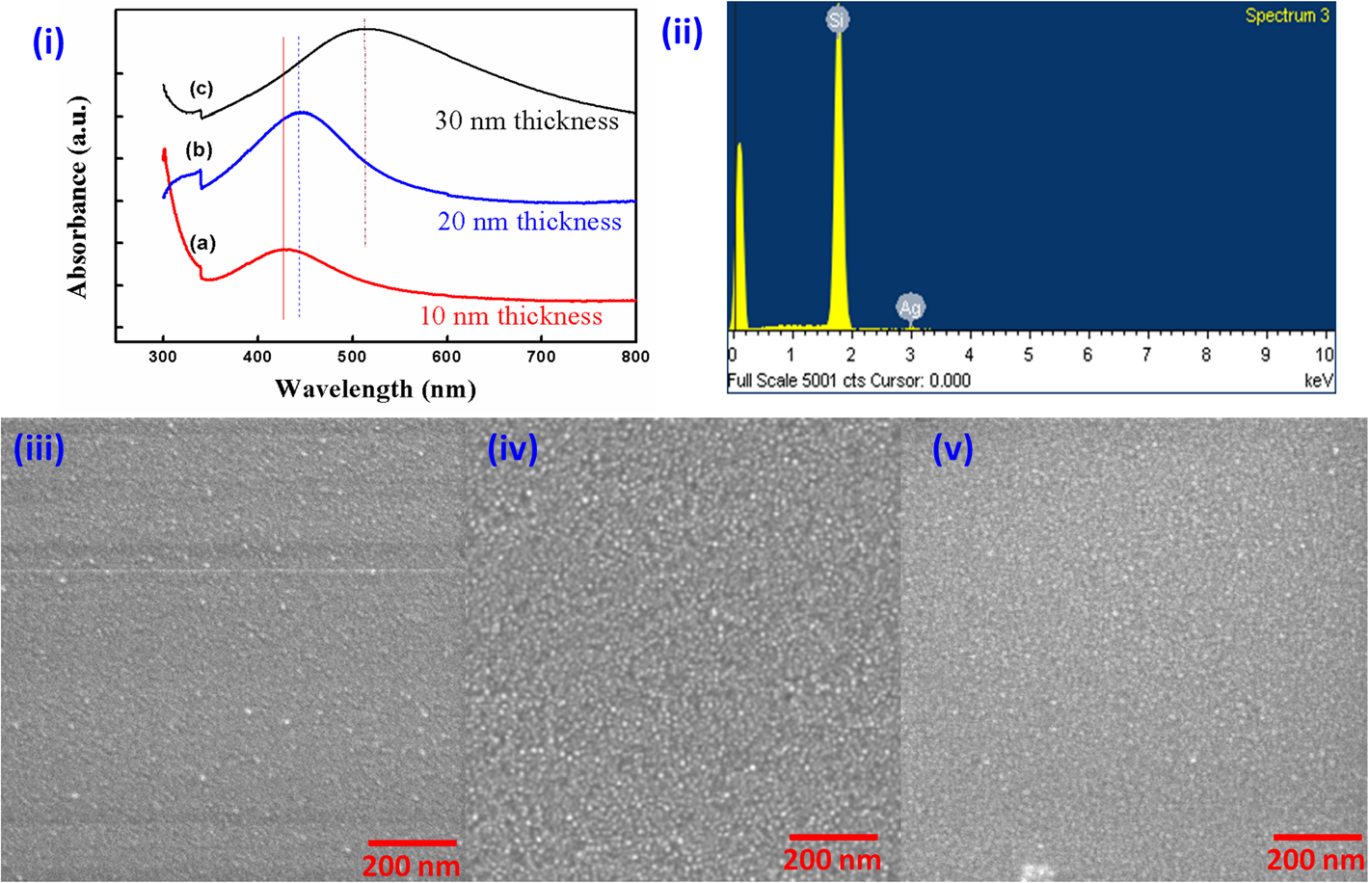
Properties of pristine Ag thin films as a function of thickness. **i** Optical absorption spectra of pristine thin films deposited on glass substrate, **ii** representative energy dispersive X-ray spectroscopy, and surface morphology corresponding to film thickness **iii** 10 nm, **iv** 20 nm and **v** 30 nm

After the film deposition, pristine films are investigated for the film’s quality verification. UV-visible absorption measurement is done to obtain optical properties of thin films prepared on glass substrates (deposited in identical conditions as on Si substrates). The optical absorbance spectra were recorded as a function of wavelength for pristine Ag films of thickness 10, 20 and 30 nm, which are shown as curves (a), (b) and (c) in Fig. 2i, respectively. The jump occurring in all spectra nearby 340 nm is due to the change of incident light source (UV lamp to visible lamp). The main characteristic peak in visible region can be understood as a result of surface plasmon resonance in nanosized Ag particles, which is in agreement to elsewhere reported optical properties of Ag nanoparticles [35–38]. In these spectra, an absorption maximum is found around 420 nm (for 10 nm thick film), whose spectral position gets red shifted to beyond 500 nm, when film thickness is increased. Further, the intensity of the absorbance peak also increases with the film thickness. Such behaviour of absorption peak can be justified on size enhancement of Ag nanoparticles with the increase in the film thickness.

EDX measurements were performed to check the elemental compositions and uniformity in elemental distribution of Ag film deposited on Si substrate. Representative EDX spectrum recorded on 30 nm pristine films is shown as Fig. 2ii. As shown in spectrum, except the peak of Si (from the substrate), there is only one significant absorption peak of Ag. The peak around 0.1 keV energy in EDX spectrum is due to lighter elements like oxygen. FESEM images of pristine Ag thin films with of 10, 20, and 30 nm thickness were recorded at 25 keV electron beam energy, as shown in the micrographs (iii), (iv) and (v), respectively, in Fig. 2. FESEM results show that Ag particles are uniformly distributed on the Si substrate and surfaces of pristine Ag thin films are found to be densely packed. Surface features of around 3–5 nm in diameter are observed in all pristine samples of different thicknesses, which can be attributed to nanocrystallite-induced roughness on the surface. The density of the nanocrystallites and size of these surface features are found to slightly increase with thickness increase.

**Fig. 3:**
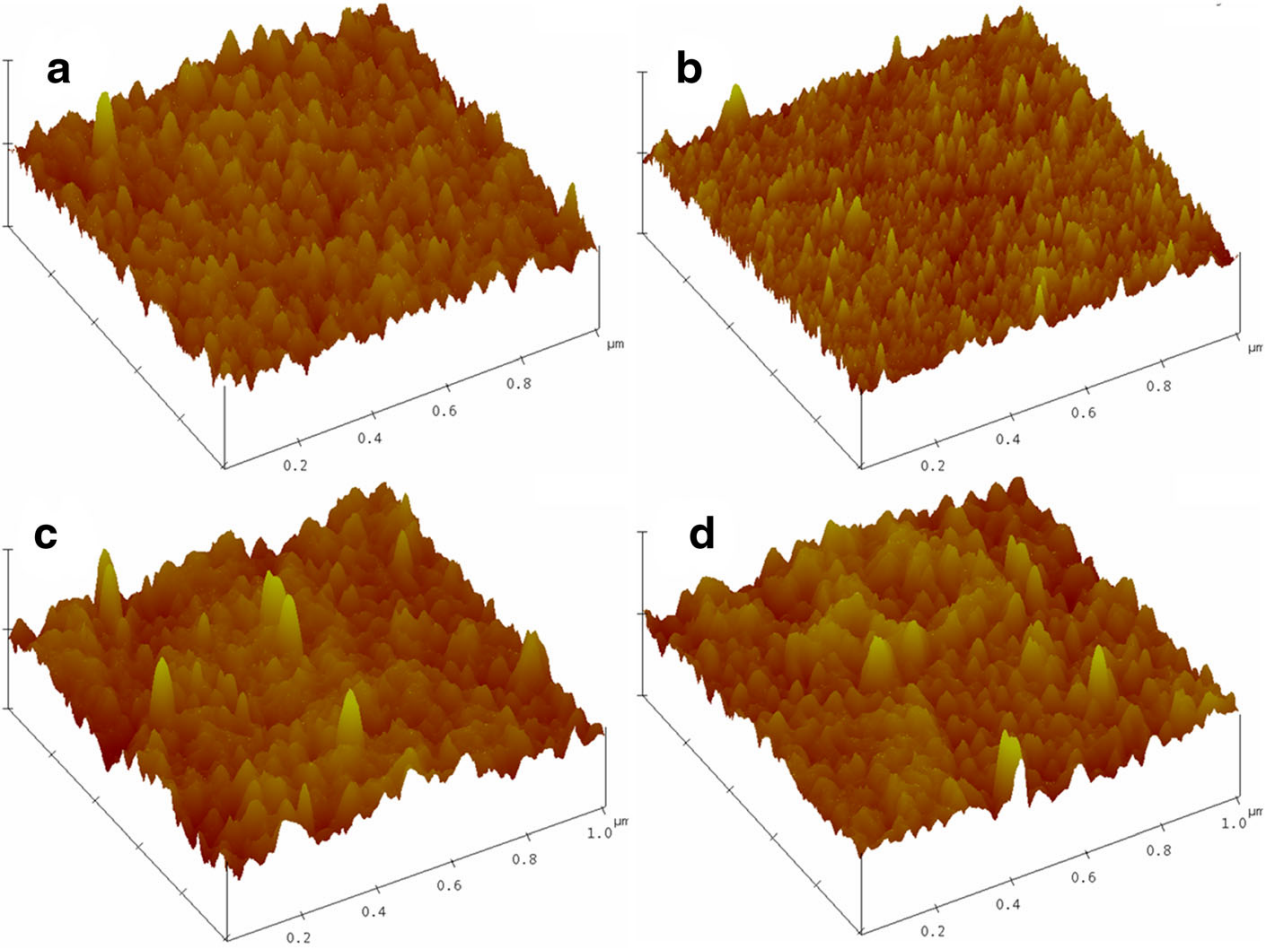
AFM imaging of 30 nm thick film **a** pristine and after irradiation using 80 keV Ar^+^ ion beam at the following fluence values: **b** 0.25 × 10^17^ ions/cm^2^, **c** 0.75 × 10^17^ ions/cm^2^ and **d** 1.0 × 10^17^ ions/cm^2^

AFM images are shown in Fig. 3 for 30 nm thick (a) pristine and two times irradiated at fluences of (b) 0.25 × 10^17^ ions/cm^2^, (c) 0.50 × 10^17^ ions/cm^2^ and (d) 1.00 × 10^17^ ions/cm^2^. From these images, it is clear that lateral size for surface features in pristine samples is ~21 nm, which is decreased to ~13 nm for the fluence of 0.25 × 10^17^ ions/cm^2^. Further, the morphology of surface exhibits cone-like nanostructures after the irradiation at fluences of 0.25 × 10^17^ ions/cm^2^ (micrograph b). In this case, the peaks of the nanostructures appeared sharp with a circular kind of base, and these features were uniform in the entire surface region. Further irradiation at higher fluence leads the higher Ag sputtering, and hence, the growth of roughness as well as uniformity in cone-like surface features are diminished. The observed values of roughnesses are found to be 1.6 nm for pristine, whereas 1.1, 1.8 and 2.1 nm for the irradiated samples at 0.25 × 10^17^ ions/cm^2^, 0.50 × 10^17^ ions/cm2 and 1.00 × 10^17^ ions/cm^2^, respectively. The results depict that a competitive process of surface diffusion and sputtering enhances the variation in height–height correlation function in the interaction zone. With the obtained AFM results, we can conclude that fluences more than 0.25 × 10^17^ ions/cm^2^ (for each time irradiation) are not appropriate for uniformity of cone-like nanostructures.

**Fig. 4:**
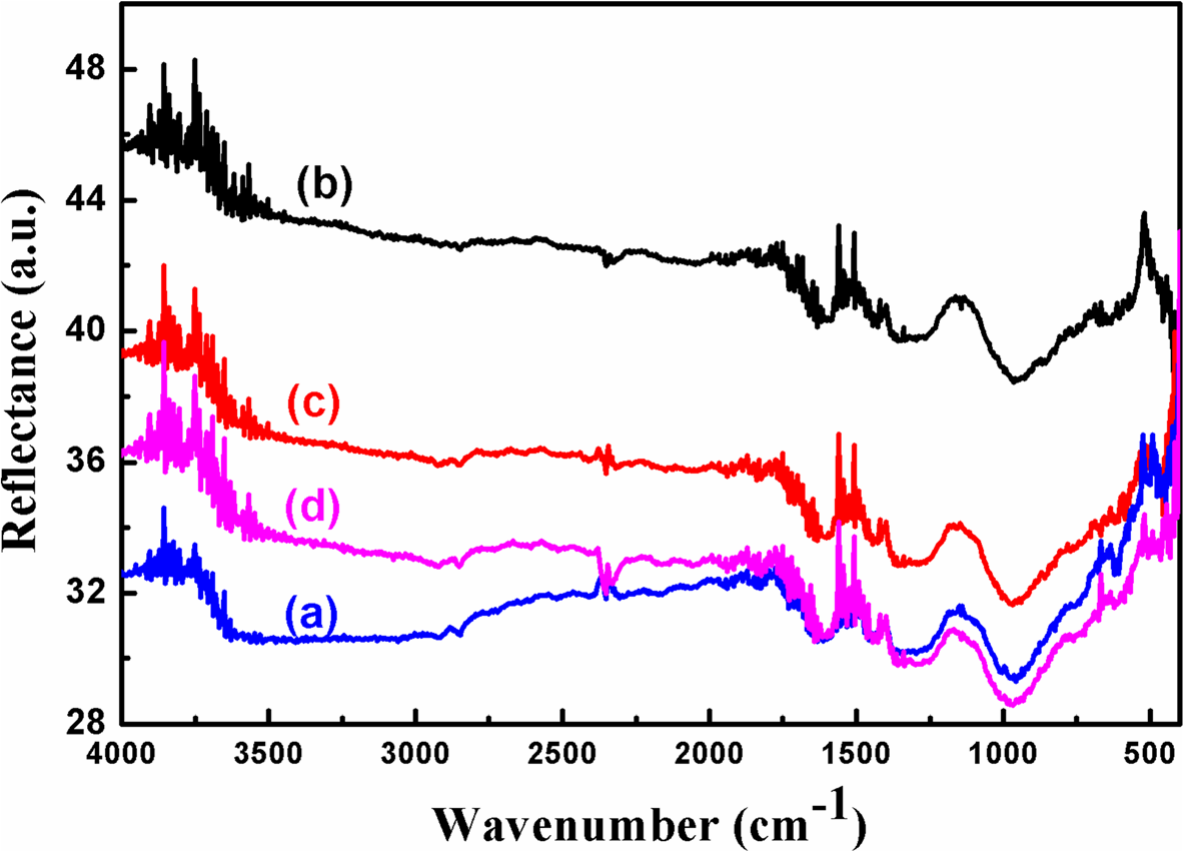
FTIR reflection spectra of 30 nm thick **a** pristine and irradiated with Ar^+^ ion beam at the following fluence values: **b** 0.25 × 10^17^ ions/cm^2^, **c** 0.75 × 10^17^ ions/cm^2^ and **d** 1.0 × 10^17^ ions/cm^2^

FTIR reflectrance properties of pristine and irradiated Ag films of 30 nm thickness are shown in Fig. 4. The plots (a), (b), (c) and (d) correspond to pristine film, and after two times irradiation at fluences 0.25 × 10^17^, 0.50 × 10^17^ and 1.0 × 10^17^ ions/cm^2^, respectively. It is observed that absorption band positions are almost the same for all the samples. Absorption bands, observed below 1000 cm^−1^, are corresponding to the stretching vibration of Si–O and Si–O–Si. Whereas, absorption bands at 952, 2850 and 2800 cm^−1^ are corresponding to Si–Si bond vibration, symmetric and asymmetric stretching of Si–H bond, respectively. Bands observed at 1300, 1610 and 2350 cm^−1^ belong to ending of O–H bond of water and CO stretching of dissolved CO_2_. Reflectance values are initially increased after the irradiation of pristine films by Ar^+^ ions (plots a and b) and then decreased after the increasing of the ion fluence (plots b–d). This implies that before irradiation, sample surface was smooth enough to reflect incident IR radiation with low divergence. Irradiation of energetic Ar^+^ ion beam with increasing fluence on the thin films initiates the removal of Ag particles from the surface, resulting in a rough surface which will scatter the incident ion beam with more divergence, and thus, reflectance values will be decreased.

**Fig. 5:**
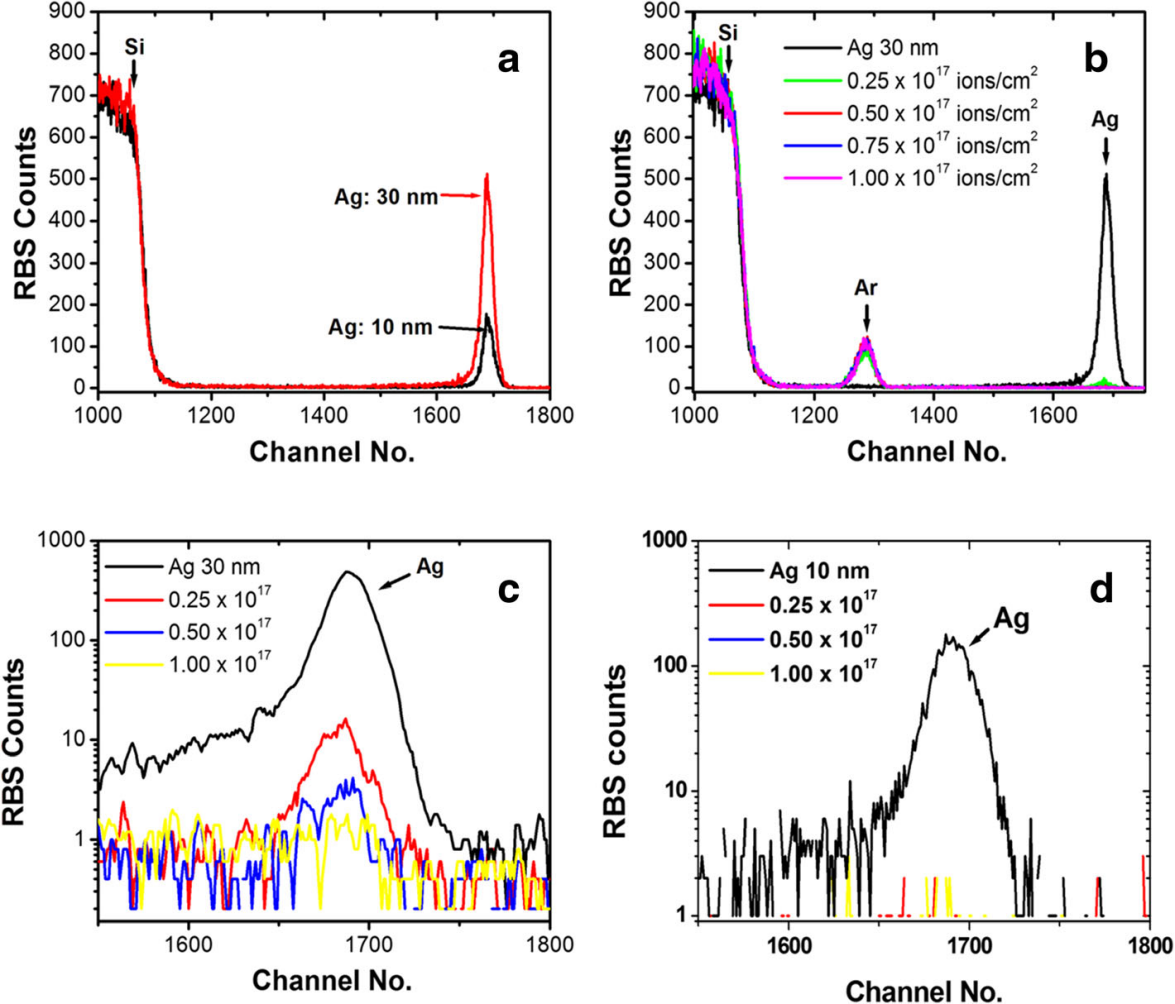
**a** RBS spectrum of pristine Ag thin films of thickness 30 and 10 nm. **b** RBS spectrum of 30 nm thickness film, before and after two times irradiation by 80 keV Ar^+^ ions at different fluence values. **c** Intensity variation of Ag signal at pristine and irradiation at different fluences for 30 nm Ag thin film. **d** Intensity variation of Ag signal at pristine and irradiation at different fluences for 10 nm Ag thin film

Experimentally observed RBS spectra for pristine and irradiated Ag thin films with the different fluence values are shown in Fig. 5. RBS spectra are obtained by plotting the normalized yield (i.e. number of counts) vs. channel number/energy. Figure 5a shows the RBS spectra of pristine Ag films of thickness 30 and 10 nm, deposited on Si. Positions of Si and Ag are indicated in figure by arrow marks. The calculation about concentration and thickness of film is performed using simulation of non-Rutherford backscattering (SIMNRA) software. Atomic concentrations of Ag for 10 and 30 nm films are estimated as 6 × 10^15^ atoms/cm2 and 18 × 10^15^ atoms/cm^2^, respectively. Choosing atomic density of Ag 5.8 × 10^22^ atoms/cm^3^, the calculated thicknesses are found as 10.4 and 31.0 nm, respectively, which is in good agreement to the thickness monitoring during the deposition of the films. RBS spectra of 30 nm thick pristine Ag thin film and after two times irradiation at the fluences of 0.25 × 10^17^ ions/cm^2^, 0.50 × 10^17^ ions/cm^2^, 0.75 × 10^17^ ions/cm^2^ and 1.0 × 10^17^ ions/cm^2^ are shown in Fig. 5b. In this figure, the positions of Si, Ag and Ar are marked. It can be seen that Ar is absent in pristine film and present in the irradiated samples. A clear vision of effect of irradiation on Ag concentration for 30 and 10 nm Ag film are shown in Fig. 5c, d, respectively. In both the cases, decrease in Ag content with increase in irradiation fluence (from 0.25 × 10^17^ ions/cm^2^ to 1.0 × 10^17^ ions/cm^2^) confirms the sputtering of Ag atoms from the surface under Ar ion bombardment. After SIMNRA simulation for 30 nm thick Ag films, the estimated values of concentration are found to be 0.7 × 10^15^ and 0.05 × 10^15^ for the fluences of 0.25 × 10^17^ ions/cm^2^ and 0.50 × 10^17^ ions/cm^2^, respectively. No Ag peak is observed for the fluence of 1.0 × 10^17^ ions/cm^2^, which indicates that the sputtering of Ag content is so high in this case that the remained content cannot be detected by RBS. In the case of 10 nm thick Ag films (Fig. 5d), the signature of Ag is eliminated at 0.25 × 10^17^ ions/cm^2^. These results confirm that sputtering induced elimination of Ag content depends on used fluence values as well as on the thickness of the film.

**Fig. 6:**
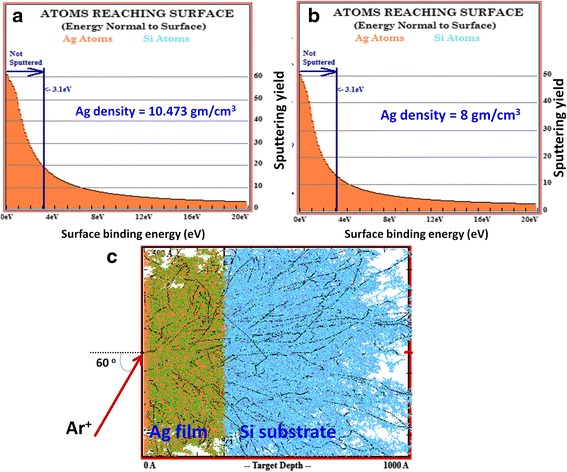
TRIM simulation plots for sputtering yield for the different density of Ag film: **a** bulk density, **b** an arbitrary low density value. **c** Energy relaxation of Ar ions in Ag thin films/Si substrate (in 100 nm × 100 nm *y*-*z* plane), after bombardment of 5000 Ar ions (in *x*-axis)

For the estimation of sputtering yield, ‘transport of ions in matter’ (TRIM) simulations were performed for 80 keV Ar^+^ ion irradiation (at 60° incident angle) on 30 nm thick Ag films deposited on Si(100) surface. For simulation, the Monte Carlo programme developed by Biersack et al. [39] was used, and 5000 ions were considered for irradiation. Lattice binding energy, surface binding energy and displacement energy for Ag were considered as 3, 2.97 and 25 eV, respectively. Considering that evaporation-induced Ag thin films can have lower density than their bulk counterpart, the integral sputtering yield was calculated for (a) bulk Ag density (10.473 g/cm^3^) and (b) an arbitrary low density value (8 g/cm^3^) as shown in Fig. 6. These plots show the energy of every recoiling atom which reaches the target surface. The average surface binding energy was found at the same value of 3.1 eV irrespective to the used density of Ag (Fig. 6 a, b). However, the number of atoms which reached the surface with more than average surface binding energy, the measure of sputtering yield, is found significantly different for the two cases (19.71 atoms/ion for (a) and 13.55 atoms/ion for (b)). This demonstrates that sputtering yield decreases with the density of target layer. The energy relaxation of Ar ions in Ag thin films/Si substrate, as shown in Fig. 6c, exhibits that the used energy parameters favour the Ar transmission into substrate. So rather confining to the role of surface treatment, present ions interact with the full volume of Ag layer and subsequently develop the obtained features.

The specific surface features are created by ion beams in two competing processes: (i) surface roughening occurring due to sputtering and (ii) smoothening due to surface diffusion. During the ion–solid interaction, the impinging low energy (~keV) ions are mostly stopped by target nuclei, because of which the energy gets dissipated over a large volume. During the passage through the target material, the incident ion transfers the significant amount of energy to the target atoms to displace them from their equilibrium position. During the collision cascade process, the ion is affected by nuclear scattering, leading to the sequence of displacement events until the recoil atom retains sufficient energy to displace other atoms. Based on the used ion species and ion energies, the governing mechanism for evolution of surface features can be different. However, the simultaneous role of surface mobility and surface diffusion both cannot be ignored. Eklund et al. reported the evolution of surface features of graphite by 5 keV Ar irradiation under the predictions of the Kardar–Parisi–Zhang equation in 2 + 1 dimensions [40]. The Bradley and Harper model [21] based on Sigmund’s theory [41] of sputtering is the most accepted approach to justify the early stage of surface morphological changes. The later stage dynamics of the morphology change is dominated by the nonlinear terms of the Kuramoto–Sivashinsky equation [42]. For the present case, considering the used fluences (in the order of 10^16^–10^17^ ions/cm^2^), such high values of sputtering yield should completely eliminate all the Ag content from the Si substrate. However, our experimental results of RBS show Ag content in 30 nm thick film up to the fluence of 0.5 × 10^17^ ions/cm^2^. Such findings can be justified by considering the dynamic changes in the local dispersion of target density and inhomogeneous surface contours during the early stages of irradiation.

Two-stage irradiation approach of solid surfaces has been used by various groups to develop new surface features and understanding of growth mechanism of such features [22, 23, 32, 34, 43]. Kumar et al. investigated the role of amorphous/crystalline (a/c) interface using the approach of sequential irradiation of pre-rippled surface of Si (100) [22, 23]. It was argued that the role of a/c interface cannot be ignored in comparison of sputtering and diffusion in surface rippling. In line with the above argument, Toyoda et al. performed the Monte Carlo simulation for cluster-ion-beam sputtering of pre-ripples surface and introduced the importance of re-deposition of sputtered atoms and diffusion toward the bottom of the valleys and hence the exponential decay of amplitudes [43]. In the work of Kim et al., related to cross-ion irradiation on pre-rippled surface of Au(001), an exponential decay of pre-patterned ripples was found and further new ripples developed only after extended flat areas form along the crossing ion beams [34]. The crossover irradiation enhanced the re-deposition of sputtered atoms due to already grown roughened surface and hence the coarsening of surface features. For the present study, the cone-like features observed at low fluence (of 80 keV Ar^+^ ions) cross-irradiation may be an effect of superposition of ripples, where sputter re-deposition and sputtering simultaneously occur. On higher fluences, sputtering of surface is much dominated than the re-deposition of sputtered atoms. It may be argued here that for the present study, the ion beam energy (=80 keV) is much higher than the one used by Kim et al. (0.5–2.0 keV). From application point of view, it should be remembered that plasmonic-based photovoltaics demands thinning of the light-absorbing layers without sacrificing the absorption, which enables more efficient carrier collection and voltage generation. The light-trapping capability of Ag nanocones is interesting in this respect and proven theoretically by full field electromagnetic simulations [44]. Owing to their tunable plasmonic response, cone-like nanostructures of Ag are reported as the promising candidate for light trapping for photovoltaics and broadband antireflection properties [44–46]. It should be further noted that Wehner et al. found micro-cones, when Cu target was irradiated by 400 to 600 eV Hg ions assisted with Mo seeding (in sputtering plasma process) [27, 47]. They stated that fairly small impurity of high sputtering yield material can promote the cone formation at the target surface. In comparison to their work (sputtering duration ~4 h and obtained microscale cone structures), the present work opens up the possibility of a fast top-down approach for cone-like metal nanostructures by low energy ion beams. We anticipate that quality of obtained cone-like nanostructures (uniformity and shape morphology) can be improved by further optimization of ion energy and using low values of fluence.

## Conclusions

This work presents a low energy ion irradiation method for cone-like Ag nanostructure formation. The pristine Ag thin films, synthesized with variable thicknesses by electron beam-assisted physical vapour deposition method, demonstrated uniform and dense deposition of Ag nanocrystallites in the range of 3–5 nm with characteristic plasmonic response. The effect of cross-grazing irradiation 80 keV Ar^+^ ions (incident at 60° to surface normal) on Ag thin films is presented as a function of ion fluence. AFM, RBS and FTIR measurements independently revealed that irradiation on Ag films are largely influenced from fluence-dependent competition between sputtering and surface diffusion. Irradiation at lower fluence are found to be appropriate for cone-like Ag nanostructures despite the large theoretical values of sputtering yields, which can be potentially used for light-trapping applications.

## Abbreviations

AFM: Atomic force microscope
EDX: Energy dispersive X-ray spectroscopy
FESEM: Field emission scanning electron microscope
RBS: Rutherford backscattering spectroscopy
SIMNRA: Simulation of non-Rutherford backscattering
TRIM: Transport of ions in matter
